# Detection of *Clostridium botulinum* group III in environmental samples from farms by real-time PCR using four commercial DNA extraction kits

**DOI:** 10.1186/s13104-018-3549-5

**Published:** 2018-07-04

**Authors:** Caroline Le Maréchal, Sarah Fourour, Valentine Ballan, Sandra Rouxel, Rozenn Souillard, Marianne Chemaly

**Affiliations:** 1 ANSES, Laboratoire de Ploufragan-Plouzané, Unité Hygiène et Qualité des Produits Avicoles et Porcins, Université Bretagne-Loire, BP 53, 22440 Ploufragan, France; 2 ANSES, Laboratoire de Ploufragan-Plouzané, Unité d’Épidémiologie et Bien-être en Aviculture et Cuniculture, Université Bretagne-Loire, BP 53, 22440 Ploufragan, France

**Keywords:** *Clostridium botulinum*, DNA extraction, Real-time PCR, Detection, Freezing

## Abstract

**Objectives:**

Few studies have tested DNA extraction methods to optimize the detection of *Clostridium botulinum* in environmental samples that can be collected during animal botulism outbreaks. In this study, we evaluated four commercial DNA extraction kits for the detection of *C. botulinum* group III in 82 various environmental samples (9 manure, 53 swabs, 3 insects, 8 water, 1 silage and 8 soil samples) collected in a context of animal botulism outbreaks.

**Results:**

The PowerSoil^®^ kit was the most efficient for almost all matrices (83.6% of the 73 tested samples), except manure for which the NucleoSpin^®^ Soil kit was the most efficient. The NucleoSpin^®^ Soil kit enabled detection in 75.3%, the QIAamp^®^ DNA Mini Kit in 68.5%, and the QIAamp^®^ Fast DNA Stool Mini Kit in 45.2%. However, the NucleoSpin^®^ Soil kit detected *C. botulinum* in 9 of the 9 manure samples tested, while the PowerSoil^®^ kit found *C. botulinum* in only two samples, and the other two kits in none of the samples. This study showed that PowerSoil^®^ can be recommended for DNA extraction from environmental samples except for manure, for which the NucleoSpin^®^ Soil kit appeared to be far more appropriate.

## Introduction

Animal botulism has increased for the last decade in Europe [1]. In France, 129 outbreaks of botulism in wild birds and 396 in poultry between 2000 and 2013 have been recorded [2]. Botulism is a paralytic disease due to the action of botulinum neurotoxin (BoNT). *C. botulinum* strains are divided into four groups based on their physiological traits and the toxins they produce. Strains belonging to *C. botulinum* group III produce type C, D, C/D and D/C toxins, which are responsible for animal botulism outbreaks.

Detection of *C. botulinum* in an outbreak context is key to identifying sources of contamination, to monitoring dissemination of the pathogen, or to validating cleaning and disinfection operations. Considering that no selective media are available, *C. botulinum* group III is detected using real-time polymerase chain reaction (PCR) after an enrichment step in anaerobic conditions. Molecular genotyping techniques, specifically PCR-based methods, are also commonly used to perform epidemiological investigations. Both approaches require good quality and yield deoxyribose nucleic acid (DNA). However, environmental samples such as dust, soil, and manure contain substances that can inhibit PCR or affect its efficiency. PCR inhibitors like humic substances, fulvic acids, polysaccharides, proteins, and organic compounds may be coextracted with DNA and affect downstream PCR application. Including a PCR inhibitor removal step to avoid these complications may therefore be necessary to obtain analyzable DNA.

Many studies have been conducted on various matrices to compare DNA extraction procedures, but most of them focus only on one matrix or one pathogen, and their efficiency in extracting DNA varied among these parameters. Extraction methods differed in their ability to recover bacterial DNA, indicating that no single DNA extraction method was optimal for all bacteria [3, 4]. Moreover, the Gram-positive, spore-forming nature of *C. botulinum* may introduce some challenges. DNA isolation from spores is considered time-consuming and arduous and sometimes uses harsh, potentially DNA-damaging chemical treatments or mechanical lysis protocols [5, 6]. An ideal commercial nucleic acid extraction kit for use with *C. botulinum* in environmental samples would provide efficient cell lysis from vegetative cells and spores, remove inhibitors and be strain-independent.

The aim of this study was to evaluate the detection of *C. botulinum* group III from various environmental samples by real time PCR using four different DNA extraction kits. The efficiency of extraction was evaluated using a *C. botulinum* group III-specific real-time PCR assay [7], which targeted *bont* genes. PCR inhibition was measured with a commercial real-time PCR inhibitor assay.

## Main text

### Materials and methods

#### Sample collection and enrichment conditions

This study included 82 samples collected from 14 different farms after a botulism outbreak (9 type C/D, 2 type D, 2 type D/C, 1 negative): 28 swabs, 25 boot swabs, 3 water pipe swabs, as well as 5 water, 1 darkling beetles, 2 fly, 1 silage, 9 manure, and 8 soil samples. Outbreaks were reported by veterinarians and farm selection was based on the owners agreeing to take part in the study.

Test samples were collected and analyzed, and contained a minimum of 20 g for soil, manure, silage, and at least 100 mL for water, and also 10–15 darkling beetles or flies. Whole samples of manure, soil and silage were half diluted in pre-reduced trypticase-peptone-glucose-yeast extract broth (TPGY), manually homogenized, and 50 g of this diluted solution was diluted five times, according to the recommendations of Standard NF EN ISO 6887-6. Water was semi-diluted in two fold concentrated pre-reduced TPGY. Swabs and boot swabs were immersed in 250 mL pre-reduced TPGY. The ends of piping swabs were prepared for testing and placed into 9 mL of pre-reduced TPGY. Flies and darkling beetles were crushed and ten fold diluted in pre-reduced TPGY. They were then homogenized for 15 s using a Pulsifier^®^ (Microgen Bioproducts, Camberley, United Kingdom). Incubation was performed at 37 °C for at least 4 days using an anaerobic chamber (Don Whitley A35, Bruz, France). After incubation, aliquots of 1 mL of each enrichment broth were prepared and stored at a temperature below − 18 °C until DNA extraction.

#### Kit selection and DNA extraction

DNA extraction was performed using four commercial kits, following the manufacturers’ instructions. The kits, using silica spin filter technology, were chosen for their easy and fast extraction methods. The following kits were evaluated: PowerSoil^®^ DNA isolation kit (Mo Bio Laboratories Inc., Carlsbad, CA, USA), QIAamp^®^ Fast DNA Stool Mini Kit and QIAamp^®^ DNA Mini Kit (QIAGEN Inc., Valencia, CA, USA), and NucleoSpin^®^ Soil (Macherey–Nagel, Duren, Germany). For NucleoSpin^®^ Soil, sample lysis was performed with the optional enhancer SX solution and SL1 buffer. In the remainder of this article, the four kits are referred to as PS, QF, QA, and NS, respectively.

Aliquots from the same sample enrichment, stored at − 18 °C, were tested. Enriched samples were stored from a few days (up to 3 days) to several months (up to 12 months). Extraction with the different kits was performed after a similar storage duration; variation in storage was only included for comparison within one kit. The sample enrichments were used for DNA extraction in duplicate using each kit.

#### Real-time PCR

The real-time PCR technique, and the primers and probes used in this study were described previously [7, 8].

#### Statistical analysis

The effect of storage time (long (up to 12 months) versus short (up to 3 days)) of enriched broth at a temperature below − 18 °C before DNA extraction on *C. botulinum* detectability was compared using a Wilcoxon test for paired samples with the software R.

### Results

#### Removal of PCR inhibitors

**Fig. 1:**
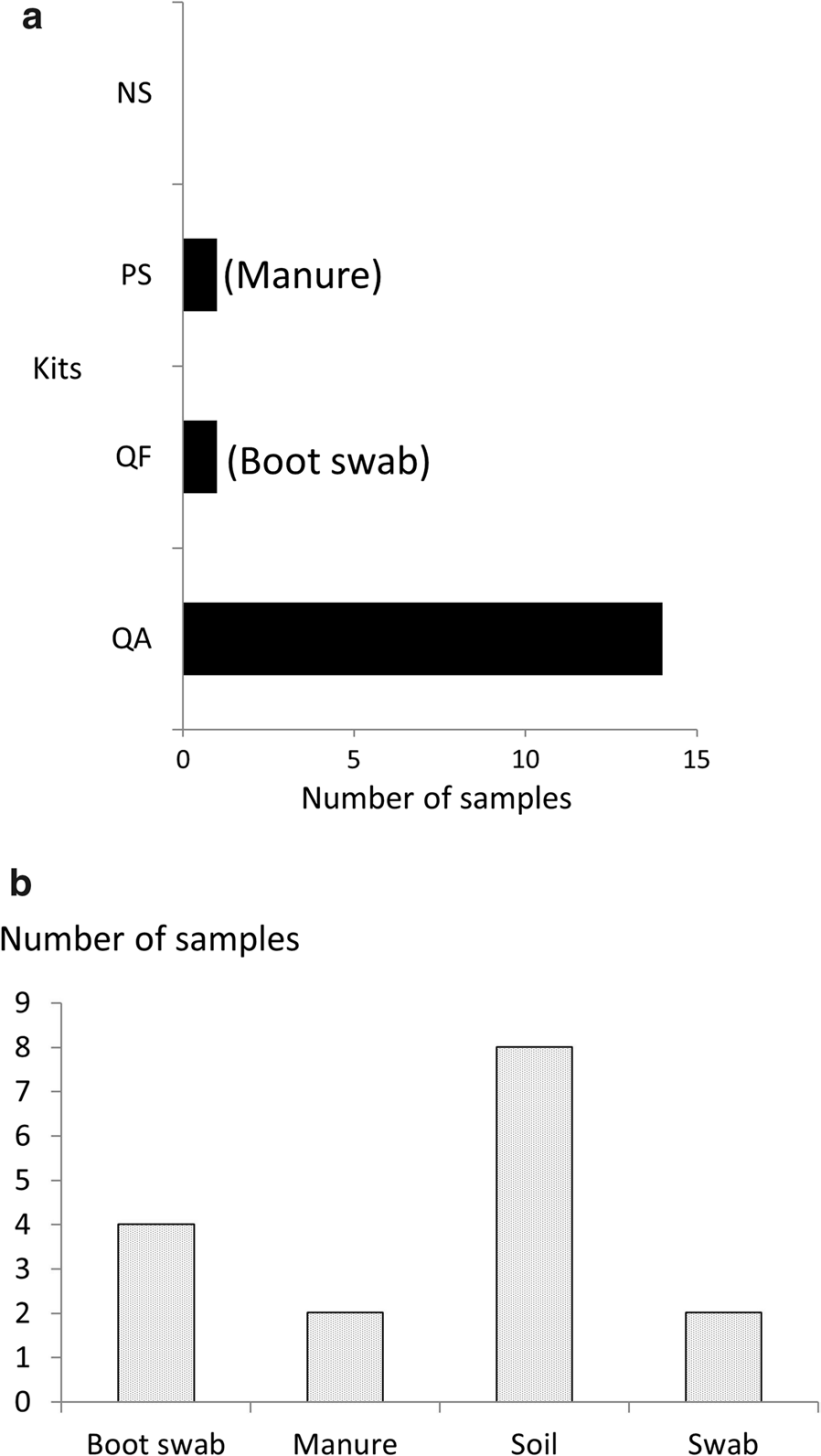
Number of samples for which the internal control was not detected. **a** According to DNA extraction kits. **b** sample types for which the internal control was not detected using the QA kit

The internal control was not detected in 14 DNA extracts when using the QA kit (Fig. 1), while the other kits enabled internal control amplification. Ten fold dilution of the DNA extracted using the QA kit allowed amplification of the internal control. More than half of the samples harboring PCR inhibitors when using QA were soils.

#### Evaluation of *C. botulinum* detection using four different DNA extraction kits

**Fig. 2:**
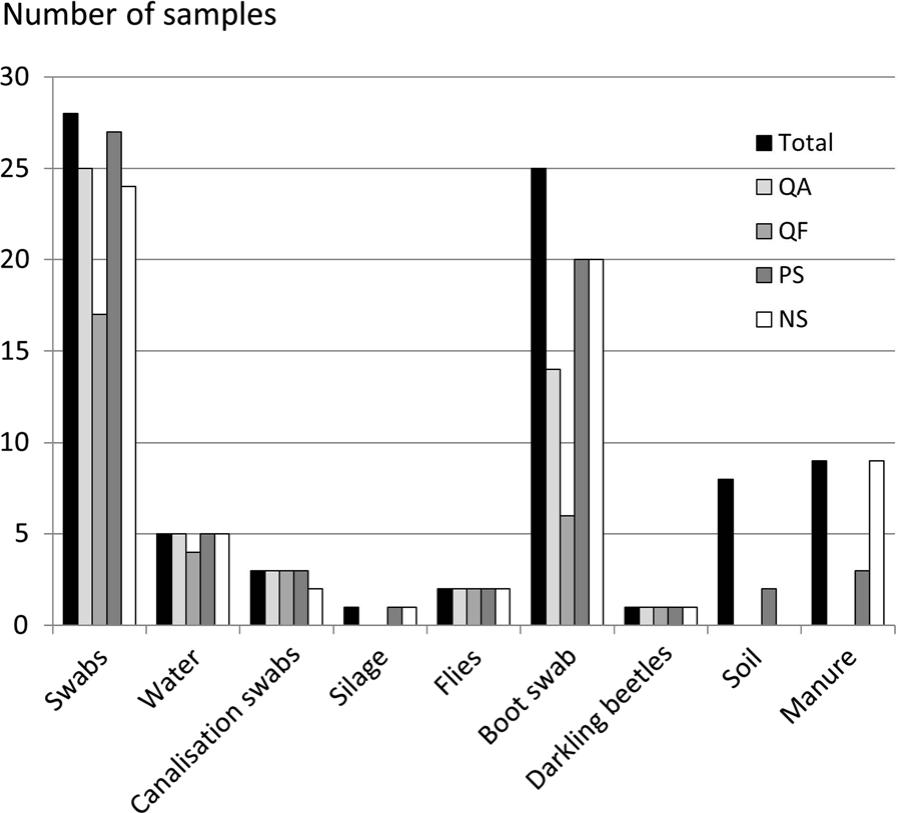
Number of samples in which *C. botulinum* group III was detected using real-time PCR according to the DNA extraction kit used and the analyzed matrix. Total: number of samples analyzed per matrix. *QA* QIAamp^®^ DNA Mini Kit (QIAGEN Inc., Valencia, CA, USA), *QF* QIAamp^®^ Fast DNA Stool Mini Kit (QIAGEN Inc., Valencia, CA, USA), *PS* PowerSoil^®^ DNA isolation kit (Mo Bio Laboratories Inc., Carlsbad, CA, USA), *NS* NucleoSpin^®^ Soil (Macherey–Nagel, Duren, Germany)

Results for *bont* gene detection are shown in Fig. 2. With the exception of manure, the PS had the best detection performance for all matrices (83.6%). The other kits that enabled detection of *C. botulinum* in 75.3% for NS, 68.5% for QA, and 45.2% for QF. Regarding detection of *C. botulinum* in manure, the NS gave the best detection results (100%). PS enabled detection in only two samples of the nine tested, while the other two kits did not detect *C. botulinum*.

#### Impact of long storage times of frozen enrichment aliquots on the efficiency of *C. botulinum* detection

**Fig. 3:**
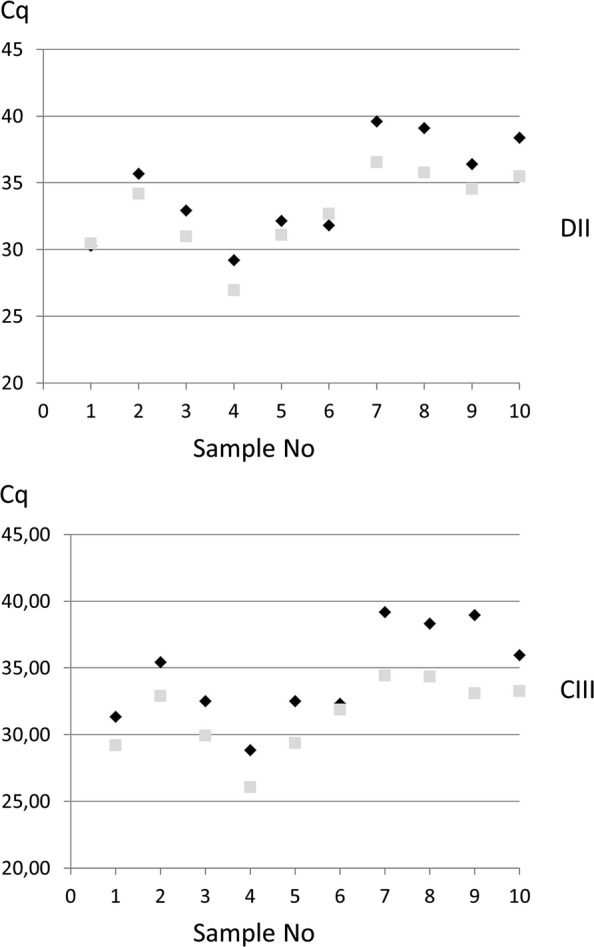
Cq values obtained with different samples from one type D/C outbreak (positive for primers and probes CIII and DII) after short (black) or long (grey) storage of the enrichment at a temperature below − 18 °C

A significant positive effect of storage at − 18 °C on the efficiency of the extraction was observed (*p * = 0.002 for CIII and 0.0098 for DII). A decrease in the Ct was indeed found after several months of storage of the enrichment at − 18 °C (Fig. 3).

### Discussion

The aim of our study was to evaluate commercial DNA extraction kits for their ability to detect *C. botulinum* group III using real-time PCR for environmental samples. The effectiveness of molecular methods is strongly influenced by DNA extraction, which may affect the sensitivity of PCR-based methods for levels of DNA yield, purity, and integrity [9].

The presence of PCR inhibitors was determined using a commercial PCR inhibition assay. The use of a dedicated PCR as an inhibition control has previously been reported, and its comparison to DNA quality using absorbance ratios showed that it provides a more accurate indication of the presence of contaminants in the DNA extract [10].

The purity of extracted DNA was evaluated using a real-time PCR assay targeting the *bont* gene Here, DNA yield or quality were not used as criteria for kit evaluation given our specific objective of detecting one targeted pathogen using real-time PCR. Likewise, the measurement of DNA quantity with different kits would not have been informative regarding the efficiency of the kits to specifically extract *C. botulinum* DNA. The enrichment medium used in our study is in fact not selective and numerous bacterial species should have grown during the enrichment step. This type of analytical approach has previously been used successfully in other studies [4, 10].

Detection rate of *C. botulinum* group III varied among kits and matrices. This result showed the relevance of evaluating DNA extraction methods for each matrix, even for a single bacterial species. Results obtained with one DNA extraction kit for one matrix cannot be systematically extrapolated to all matrices. Similar differences between substrate types (soil and feces) or even subtypes (depending on the soil sample characteristics) and DNA extraction methods have previously been reported [10–12].

Only one soil sample was detected positive among all the samples tested (PS). This could be explained by the fact that coextracted charged organic compounds such as humic acids compete with nucleic acids for silica-binding sites, causing much of the nucleic acids to pass through. In addition, inhibitors present in the extract may also bind to the nucleic acids, preventing their retention on the silica filter [13]. Absence of amplification when using QA for soil DNA extraction might show that PCR inhibitors were not removed or were coextracted when using this kit.

The PS has been reported several times as being a good DNA extraction kit for different pathogens and matrices, either for DNA yield or for PCR inhibitor removal [11, 14, 15]. This seems to be consistent with our results.

The QF appeared to be the least appropriate for DNA extraction for *C. botulinum* detection in environmental samples. Such results have already been reported for other pathogens, specifically in pig manure [12] or feces [4] when using the QIAamp DNA Stool Mini Kit. This could be explained by the absence of a mechanical disruption step, which is done with the PS and NS kits. Bead beating methods have been reported to be more efficient than chemical methods in detecting genes of various Gram-positive bacteria present in human fecal samples and sludge samples [16, 17]. Surprisingly, QA gave better results than QF, although it is not recommended for complex samples. QA was previously shown to be the best extraction method for *C. botulinum* detection in various matrices when compared to three other methods [9].

NS was reported to be the best kit in another kit comparison study conducted on pig manure or lagoon effluent [12].

Long time storage of frozen enrichment aliquots significantly decreased the Ct in our study. Some other studies have suggested that freezing might slightly improve DNA extraction from selected Gram-positive bacteria [18]. It has also been shown that when expressing the bacterial data as relative abundance, the proportion of *Clostridium* in total bacteria was higher (*p* < 0.05) in frozen stored feces than in fresh feces [19].

### Conclusion

The evaluation of four different commercial DNA extraction kits showed that the PowerSoil^®^ DNA isolation kit was the most appropriate to extract DNA for the detection of *C. botulinum* group III in enriched environmental samples, with the exception of manure samples for which the NucleoSpin^®^ Soil kit yielded better results. In this study, we identified the appropriate kits for the detection of *C. botulinum* group III in environmental samples for various matrices. We also showed that long storage (several months) of enriched broth before DNA extraction improves the detectability of *C. botulinum* group III. This will be very helpful for epidemiological investigations and in understanding initial contamination during animal botulism outbreaks.

## Limitations

Results obtained here are only valid for the detection of *C. botulinum* group III and the matrices tested here after enrichment of samples according to the conditions described in our study. The evaluation of the DNA extraction kits was only performed using naturally contaminated samples collected in botulism outbreaks context (with no information on the effective contamination or the amount of spores/bacteria in the samples) and not using spiking samples or using samples with a proved contamination, as commonly performed for DNA extraction kit comparison. Such evaluation should be performed again for other pathogens, other conditions of sample processing or a new matrix. The effect of long storage (several months) at a temperature below − 18 °C on the detection of *C. botulinum* group III was tested on a small number of samples. However the difference between short storage and long storage on the detection was significant and appeared so worth to be included in this article.

## Abbreviations

BoNT: botulinum neurotoxin
DNA: deoxyribose nucleic acid
NS: NucleoSpin^®^ Soil (Macherey–Nagel, Duren, Germany)
PCR: polymerase chain reaction
PS: PowerSoil^®^ DNA isolation kit (Mo Bio Laboratories Inc., Carlsbad, CA, USA)
QA: QIAamp^®^ Fast DNA Stool Mini Kit
QF: QIAamp^®^ DNA Mini Kit (QIAGEN Inc. Valencia, CA, USA)
